# Biochemical and Physiological Performance of Seeds of *Pentaclethra macroloba* (Willd.) Kuntz (Leguminosae, Caesalpinioideae) at Different Phases of Maturation

**DOI:** 10.3390/plants14071112

**Published:** 2025-04-02

**Authors:** Olívia Domingues Ribeiro, Reynaldo Azevedo Santos, Mário Augusto Gonçalves Jardim, Jaisielle Kelem França Benjamim, Thiara Luana Mamoré Rodrigues Hirosue, Eloisa Helena de Aguiar Andrade, Mozaniel Santana de Oliveira, Ely Simone Cajueiro Gurgel

**Affiliations:** 1 Botany Coordination, Museu Paraense Emílio Goeldi, Av. Perimetral, 1901, Terra Firme, Belém 66077-830, Braziljardim@museu-goeldi.br (M.A.G.J.); mozaniel.oliveira@yahoo.com.br (M.S.d.O.);; 2 Graduate Program in Biodiversity and Biotechnology—Rede Bionorte, Av. Perimetral, 1901, Terra Firme, Belém 66077-830, Brazil; thiaramamore@gmail.com

**Keywords:** amazon, bioeconomy, germination, oil yield, pracaxi, sustainable extractivism, unsaturated fatty acids

## Abstract

Determining the optimal harvest time for *Pentaclethra macroloba* seeds is essential to preserve germination potential and ensure high-quality oil production, valued in the pharmaceutical and cosmetic industries. This study aimed to identify the maturation phase that maximizes seed physiological quality and oil yield. Fruits and seeds were collected from 44 mother plants in Belém and São Domingos do Capim, Brazil, during three final maturation phases: P1 (dark green pericarp, beige seeds), P2 (yellowish-green pericarp, light-brown seeds), and P3 (black pericarp, dark brown seeds). Germination, vigor tests, and gas chromatography analyses revealed that seeds from P3 exhibited the highest vigor (93–99% germination) and oil yield (up to 13.1%). Major fatty acids were oleic (up to 65.23%), linoleic (up to 8.45%), and behenic acids (up to 17.22%). The ripening period ranged from 7 to 8 months, influenced by environmental factors. Optimal seed quality and oil yield are achieved when harvesting occurs before dispersal, targeting fruits with yellowish-green pericarp transitioning to black. Post-harvest drying enhances oil extraction efficiency. These findings support the conservation of *P. macroloba* through viable seeds and promote economic sustainability by optimizing oil production, benefiting biodiversity and local extractive communities.

## 1. Introduction

*Pentaclethra macroloba* (Willd.) Kuntze (Leguminosae, Caesalpinioideae), commonly referred to as pracaxi [1], is widely distributed throughout South America and parts of Central America [2]. In Brazil, the species is found in the states of Acre, Amazonas, Amapá, Pará, and Roraima [3]. It is typically found in flooded areas, gallery forests [4,5], and terra-firme forests [6].

The presence of species adapted to flooding, such as *P. macroloba*, is of great ecological importance. These species play a vital role in the stabilization of riverbanks, the reduction of erosion, the improvement of water quality, and the regulation of the hydrological cycle. Furthermore, they function as a carbon sink, thereby contributing to climate change mitigation [7,8].

The species has an average height of 13 m within the Amazon estuary [9]. The trunk is either straight or sloping, cylindrical in shape, with a rough bark and legume-type fruits that are desiccated. The fruits are green in color when immature and darken in hue as they reach maturity, containing two to seven seeds per fruit [10]. Once the seeds have reached maturity, they are dispersed between the months of January and April, which coincides with the period of highest rainfall in the Amazon region [11].

The *P. macroloba* plant begins to flower and bear fruit one to two years after germination. The mature seeds are dark brown and exhibit pleurograms [10,12]. In the Amazon region, dispersal is discontinuous and irregular [4], occurring primarily through autochoric means, specifically explosive dehiscence, and secondarily through hydrochory, with seeds transported by waterbody [9]. However, fruit and seed production varies significantly between individual plants [10].

Non-timber forest products (NTFPs) represent an alternative source of income for traditional communities, as they contribute to biodiversity conservation and stimulate the local economy [13]. *P. macroloba* possesses the potential to serve as an NTFP, with the extraction of fixed oil from its seeds providing economic benefits for local communities and contributing to the conservation of the species and its habitat, provided that the extraction is carried out in a sustainable manner [9].

*P. macroloba* seeds contain a high fixed oil content, with a yield of up to 51% [12]. It is characterized by a light-yellow hue and is reputed to possess healing properties, which have been demonstrated to be effective in the treatment of a range of ailments, including muscle pain, inflammation, and infections [12,13]. The oil’s chemical composition is noteworthy for its bactericidal elements [14]. The high concentration of white fat in this substance renders it suitable for soap production and candle making, as it remains in a liquid state at room temperature [15]. Moreover, it can be utilized as an alternative raw material for biofuel production [16].

The remarkable properties of the oil are closely linked to the physiological and biochemical changes that occur during seed maturation [17,18]. Maturation is the consequence of the numerous alterations that take place in a seed during its development. These changes include an increase in size, variation in water content, vigor, and the accumulation of dry matter [19]. The development of seeds occurs in distinct phases, commencing with an augmentation in water content. This is subsequently followed by cell expansion, which results in an increase in size and the deposition of reserves [20].

An understanding of the phases of fruit and seed development is of paramount importance for the effective production of seedlings and the harvesting process. The evolution of these phases is subject to climatic variations specific to each region [21,22]. It is, therefore, essential to assess the phase of maturation of *P. macroloba* seeds in order to determine the optimal time for harvesting the fruit. The traditional method of collection is conducted after the seeds have been dispersed, either on the beach sand during low tide or in rivers. This is achieved through the use of canoes and panniers for the collection of seeds in the water [13,23]. Subsequently, seeds are sorted to discard those with undesirable characteristics, including dark coloration, soft texture, and evidence of gnawing [23].

The successful production of seedlings and oils from native forest seeds is contingent upon the implementation of an appropriate collection, storage, and extraction process that safeguards the viability of seeds and the chemical properties of the species’ fats [24]. The objective of this study was to determine the optimal ripening phase for obtaining *Pentaclethra macroloba* (Willd.) Kuntze seeds exhibit the highest physiological potential and the highest extracted oil quality.

## 2. Results and Discussion

### 2.1. Ripening Variability and Seeds Water Content

The fruits harvested in Belém and São Domingos do Capim (SDC) exhibited a longer ripening period than the six months documented by Dantas et al. [10], who identified seven phenophases. The ripening period exhibited variation between the two collection sites, with a duration of seven months in Belém and eight months in SDC, underscoring the heterogeneity in the ripening process among *P. macroloba* populations. This variability was attributed to environmental factors, such as soil conditions and microclimatic variations, which significantly influence seed development and the maturation process [25].

The várzea forest, where the collections were made, displays notable variations in environmental characteristics between sites. While SDC is regarded as a less disturbed environment, Belém is in a region with high anthropogenic impact [26,27]. These environmental distinctions have the potential to influence the maturation process of fruit and the viability of the resulting seeds. The unevenness of ripening may also result from interactions between environmental and genetic factors, which are closely associated with phenological events affecting flowering and fruiting processes [28]. The influence of elevated CO_2_ levels on fruit ripening, as previously discussed by Krupa and Tomala [29], lends further support to the notion that local environmental conditions, including microclimatic variations, have the capacity to alter physiological parameters such as fruit softening and soluble solids content. The observed heterogeneity is likely attributable to the distinct microclimatic and soil conditions present at the two sites.

The water content of *P. macroloba* seeds, derived from specimens procured from Belém and SDC, exhibited the following values: 41.84% and 47.66% in P1, 35.96% and 38.91% in P2, and 16.60% and 19.88% in P3, respectively. The water content of the seeds was observed to decrease as the fruit progressed through the ripening phases, regardless of the location from which the fruit was obtained.

As documented in the literature, the water content of seeds from a range of plant species, including legumes such as *Anadenanthera colubrina* (Vell.) Brenan, undergoes a notable decline during the final phase of development [30]. Similarly, the same phenomenon is observed in *Sesbania virgata* (Cav.) Poir. [31] and *Saraca asoca* (Roxb.) De Wilde [32] begins the ripening process with a high water content, which subsequently decreases as the maturation progresses. Furthermore, both *Arachis hypogaea* L. [33] and *Parapiptadenia rigida* (Benth.) Brenan [34] undergo a gradual reduction in water content as the seeds mature. As the seeds accumulate nutrients and reserve substances, the water is gradually removed, contributing to an increase in dry matter mass [19].

### 2.2. Physiological Quality of Seeds in the Three Phases of Maturation

The seed emergence onset (ISE) was observed to be 27.5 and 21.75 (P1), 24.5 and 18.5 (P2), and 17 and 12 (P3). The mean emergence time (AET) was 28.76 and 24.09 days (P1), 27.20 and 26.05 days (P2), and 22.91 and 17.46 days for seeds from P3. The germination rate (G) exhibited considerable variation, with values ranging from 23% to 37% (P1), 41% to 61% (P2), and 93% to 99% (P3) in Belém and SDC, respectively (Table 1 and Table 2).

The results demonstrated that maturation exerted an influence on all variables tested in both the Belém and SDC locations (Table 1 and Table 2), except for dead seeds (DS), which exhibited no significant correlation with maturation in the SDC location (Table 2). The lack of significant differences in DS for SDC may be attributed to the low incidence of dead seeds in the collected samples, resulting in insufficient variability to detect statistical differences. Therefore, the data for DS in SDC were not included in further analyses. The seeds of P3 differ from those of P2 and P1 in terms of ISE, SEI, AET, E, US, G, EL, RCD, RDM, and APMD.

The germination test demonstrated that the seeds in the three maturation phases were viable for germination. Nevertheless, the seeds in P3, which represent the final phase of maturation of *P. macroloba* fruits, exhibited a higher germination percentage. The seeds germinated rapidly and uniformly, resulting in a shorter average time to emergence when compared to the seeds in the other phases. This performance was observed in the ISE, SEI, and AET values. The seeds in the P1 and P2 phases required a longer period to germinate, indicating that the maturation phases exert an influence on these variables. These findings indicate a notable correlation between the maturation phase of *P. macroloba* seeds and their germination capacity, with seeds in P3 exhibiting superior performance compared to those in other phases.

A number of species belonging to different families exhibited markedly elevated germination potential in the final phase of maturation. Examples include *Allophylus edulis* (A.St.-Hil.) Hieron. ex Niederl. (Sapindaceae) [35], *Parapiptadenia rigida* (Benth.) Brenan (Leguminosae) [35], *Lophantera lactescens* Ducke (Malphigiaceae) [36], *Anandenanthera colubrina* (Vell.) Brenan (Leguminosae) [30], and *Physalis ixocarpa* Brot. ex Hornem. (Solanaceae) [37].

It is important to note, however, that the study of maturation holds pertinence in certain species due to their unique characteristics, such as the variation in seed maturation patterns and the subsequent impact on germination and viability. Seeds demonstrate the capacity to germinate during the initial phases of maturation [19,30]. Nevertheless, the seeds may fail to produce normal and vigorous seedlings because of the immaturity of the embryo [33].

It is not uncommon for certain species to reach physiological maturity prior to the designated point of harvest. As observed in *Capsicum baccatum* var. *pendulum* (Willd.) Eshbaugh [38], *Physalis angulata* L. [39], and *Saraca asoca* (Roxb.) Willd. [32], which are all members of the Solanaceae family, as well as in the Leguminosae family, exemplified by *Saraca asoca* (Roxb.) Willd. Moreover, in instances such as those observed in *Glycine max* (L.) Merr. [40] and *Arachis hypogaea* L. [33], the seeds continue to enhance their viability even after reaching physiological maturity. The diversity of maturation patterns underscores the intricate nature of seed development processes, necessitating comprehensive investigations to elucidate these mechanisms across diverse species.

The seedlings derived from P3 seeds exhibited enhanced vigor, as evidenced by the indices of days to initial seedling emergence, average emergence time, and speed of emergence index. The seedlings derived from P1 and P2 exhibited diminished vigor in both collection areas. These differences were also reflected in the percentage of dry matter mass in the roots and aerial part, which was higher in seedlings from P3. The findings indicated that, for *P. macroloba*, the advanced phase of maturation (P3) resulted in enhanced performance, exerting a beneficial influence on the vigor variables of the seedlings. Therefore, seeds from P3 can be utilized to produce robust seedlings (Table 1 and Table 2).

The findings of this study were corroborated by those of similar investigations conducted on seeds of other Leguminosae species, including those of *Inga striata* Benth. [41]: *Mimosa caesalpiniifolia* (Benth.) [42]; *Inga laurina* (Sw.) Willd. [43]; and *Albizia niopoides* (Spruce ex Benth.) Burkart [44] all exhibited enhanced germination potential and seedling vigor when compared to immature seeds.

The results of this study suggest that the physiological potential of *P. macroloba* seeds increases as the maturation phase progresses. This phenomenon was corroborated by the significant presence of US seeds (seeds that did not commence the imbibition process and appeared to be intact) and the negligible occurrence of dead seeds and abnormal seedlings identified in the P1 and P2 phases at both collection sites. It is noteworthy that *P. macroloba* seeds lack tegumentary dormancy [45], indicating the absence of a physical barrier to water entry. However, a considerable proportion of US seeds could be attributable to hormonal imbalances, as posited by Baskin and Baskin [46], who advanced the notion that such imbalances might impede the optimal germination process in specific species. It seems plausible to suggest that the embryos of the P1 and P2 seeds were immature, which may necessitate a longer germination period. Seeds that are immature and recalcitrant often exhibit delayed germination due to a lack of embryo maturity [47].

Significant differences were observed between P1 and P2 seeds for several variables, including ISE, AET, US, ED, RDM, and APDM, in both locations. The results indicate that P2 exhibits a higher physiological potential than P1, suggesting that P2 is approaching the end of the maturation process (Table 1 and Table 2).

### 2.3. Yield and Chemical Composition of Oils

The oil content of *P. macroloba* seeds in P3 was found to be significantly higher than in P2, with an increase of approximately 32% in Belém and 42% in SDC. This evidence suggests that seed oil content is influenced by maturity (Table 3).

The timing of the harvest had a significant impact on the yield of *P. macroloba* seeds, with a gradual increase in oil content observed as the fruits matured. The most favorable outcomes were observed in black fruits and seeds with dark brown seed coats. These patterns have also been observed in other species, including *Jatropha curcas* L. (Euphorbiaceae) and *Glycine max* (L.) Merr. (Leguminosae). In these cases, oil content was found to increase towards the end of the ripening process. However, in canola (*Brassica napus* L., Brassicaceae) [48], there was a reduction of approximately 10% in oil content in the final phase of ripening. These findings underscore the significance of fruit and seed morphological characterization for identifying the optimal harvesting period.

Table 4 shows the fatty acid compositions of the oils extracted from *P. macroloba* seeds in the three maturation phases investigated, with the identification of 12 acids.

The chemical composition of *P. macroloba* oil after transesterification was consistent with the patterns previously identified by other researchers [49,50]. These studies identified oleic (ω9), behenic, lignoceric, and linoleic (ω6) acids as the predominant constituents of the oil.

No correlation was identified between the fatty acid profile and the seed maturation phases. However, differences were observed in the percentages of these acids in relation to the collection sites. The oils extracted from Belém seeds exhibited higher percentages of oleic (ω9), lignoceric, and linoleic (ω6) acids, whereas behenic acid was more concentrated in all phases of SDC. Furthermore, tricosylic and nervosol acids were exclusively identified in SDC samples. These differences in the composition of fatty acids and oil content in the seeds highlight the complexity of the interactions between genetic and environmental factors, which ultimately give rise to diverse responses of plant populations to environmental conditions [51].

**Figure 1:**
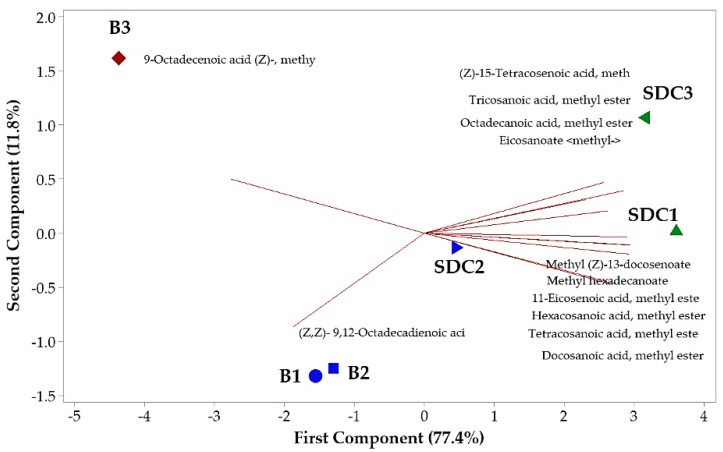
Biplot (PCA) resulting from the analysis of identified components in the oil seeds of *Pentaclethra macroloba* (Willd.) Kuntze. Legend: Location and maturity phase—(B1, B2, B3: seeds from Belém at maturity phases 1, 2, and 3, respectively); (SDC1, SDC2, SDC3: seeds from São Domingos do Capim at maturity phases 1, 2, and 3, respectively). Matching colors denote similarity among groups.

The principal component analysis (PCA) revealed the formation of three distinct groups, with the first two components accounting for 89.2% of the total variance (Figure 1). PC1 was strongly influenced by oleic and behenic acids, while PC2 was more related to linoleic and palmitic acids. The clear separation of groups reflects significant differences in fatty acid composition across maturation phases, with P3 samples from Belém showing a distinct profile characterized by higher oleic acid content. These findings suggest that P3 is the most suitable phase for oil extraction, particularly for applications requiring high oleic acid content.

**Figure 2:**
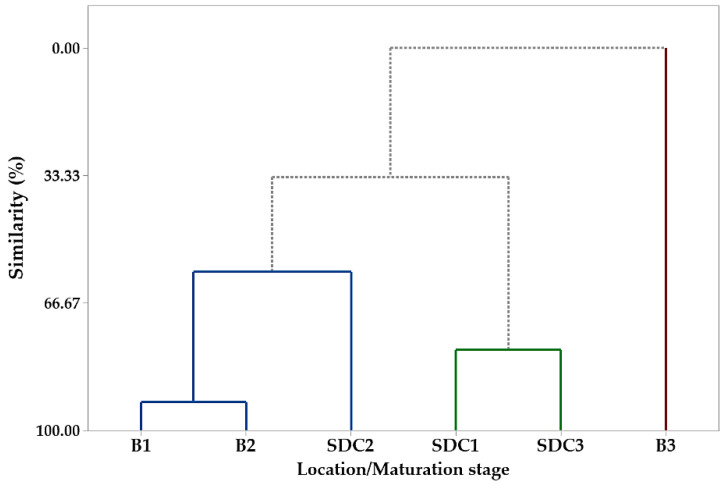
Dendrogram representing the similarity relationship resulting from the analysis of the compounds identified in the oil seeds of *Pentaclethra macroloba* (Willd.) Kuntze from different locations and maturity phases: B1, B2, and B3 (seeds from Belém at maturity phases 1, 2, and 3, respectively); SDC1, SDC2, and SDC3 (seeds from São Domingos do Capim at maturity phases 1, 2, and 3, respectively). The colored lines represent clusters of similarity between samples based on their chemical composition, grouping seeds by location and maturity phase.

The hierarchical cluster analysis (HCA) corroborated the PCA results, forming three groups based on fatty acid composition (Figure 2). Group I included P1 and P2 samples from Belém and P2 from SDC, characterized by higher linoleic and behenic acid concentrations. Group II comprised P1 and P3 samples from SDC, with elevated levels of palmitic and stearic acids. Group III consisted exclusively of P3 samples from Belém, distinguished by their high oleic acid content. These results align with previous studies on *P. macroloba* oil composition [50] and highlight the influence of both the maturation phase and collection site on fatty acid profiles.

The hierarchical cluster analysis (HCA) revealed the formation of three distinct groups comprising the different samples. Group I was constituted of samples S1, S2, and S5, which were extracted from Belém seeds (S1 and S2 phases P1 and P2, respectively) and SDC (S5, P2). Group II was constituted by samples S4 and S6, which were collected in SDC in P1 and P3, respectively. Group III was constituted by sample S3, which was collected in Belém in P3. The formation of the groups was influenced by certain compounds. For instance, linoleic acid, behenic acid, and lignoceric acid were instrumental in the formation of group I. In group II, the compounds that played a pivotal role in its formation were nervosonic acid, tricosylic acid, stearic acid, arachidic acid, palmitic acid, erucic acid, cerotic acid, and gondoic acid. Group III was solely composed of oleic acid.

The three maturation phases analyzed at the two collection sites exhibited high concentrations of unsaturated fatty acids. P3 from Belém exhibited a distinctive fatty acid profile, comprising 73.36% of the total composition. Of this, 65.23% was oleic acid (ω9), and 6.68% was linoleic acid (ω6), an essential fatty acid. Although oleic acid is not an essential nutrient, its consumption has been linked to a reduction in the incidence of cardiovascular diseases, improved secretory activity of the pancreas and liver, a decreased risk of gastric-duodenal ulcers, and an important role in brain development [52]. Likewise, linoleic acid, a polyunsaturated essential fatty acid, cannot be synthesized by the body and plays a pivotal role in maintaining healthy levels of triglycerides and cholesterol [53].

The oil of *P. macroloba* exhibits a higher concentration of oleic acid in comparison to other species, including industrial and native Amazonian varieties such as soybean (*Glycine max*) (20.48%) and sunflower (*Helianthus annuus* L., Asteraceae) (21.93%) [54]. Additionally, the oil of *P. macroloba* displays a higher concentration of oleic acid than that of the Brazil nut (*Bertholletia excelsa* Bonpl.). Additionally, the oil of Lecythidaceae (41.62%) [55], *Lecythis pisonis* Cambess. (Lecythidaceae) (48.10%) [56], *Euterpe oleracea* Mart. (Arecaceae) (37.4%) [57], and *Carapa guianensis* Aubl. (Meliaceae) (44.87%) [57] have also demonstrated noteworthy concentrations of beneficial fatty acids. Moreover, *P. macroloba* oil is distinguished by its elevated concentration of behenic acid, a distinctive attribute that is not observed in the species. The use of behenic acid is pervasive in industrial applications due to its gelling properties, which are instrumental in enhancing the stability and mechanical characteristics of gels. These properties are of particular significance in the commercial development of oleo gels in cosmetic and pharmaceutical products [58].

The results of the seed viability and oil yield analyses conducted across the three maturation phases indicated the presence of a maturation phase between phases P2 and P3. This is because P3 has the shortest period of permanence on the plant among the phases that were analyzed. In this phase, the fruits underwent desiccation, thereby preparing for seed dispersal. The seeds harvested in P2 exhibited a higher water content, approximately 19% greater than that observed in the seeds of P3. This finding corroborates the existence of the proposed new phase. During field observation, it was noted that the fruits, which were initially yellowish-green in color, began to darken, acquiring a black hue. Additionally, the seeds exhibited a light brown coloration in the center and a dark brown hue at the ends.

The transition point represents the optimal time for harvesting the fruits of *P. macroloba*, allowing for their harvesting and subsequent drying to complete the desiccation process. This practice facilitates large-scale harvesting with lower water content, preventing the spontaneous opening of the fruits and ensuring greater physiological potential. Furthermore, it results in superior yield and quality in oil extraction.

## 3. Material and Methods

### 3.1. Fruit Collection Area

**Figure 3:**
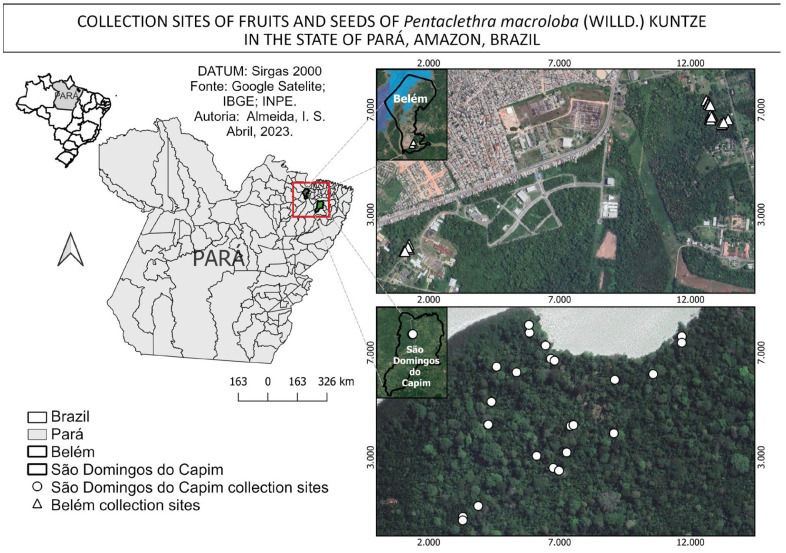
Areas and collection points for fruits and seeds of *Pentaclethra macroloba* (Willd.) Kuntze in the state of Pará, Amazonia, Brazil.

The fruits were procured from 44 mother plants over the course of two months, from December 2022 to January 2023. The collection was conducted in an alluvial dense ombrophilous forest, which is locally known as a várzea forest. The forest is situated within the administrative boundaries of the municipalities of São Domingos do Capim [27] and Belém [26]. The latter is situated in an area with a high level of anthropogenic impact, as it is within the campus of a federal university. The former is situated in an area with a relatively lower degree of anthropogenic impact, as illustrated (Figure 3).

### 3.2. Collecting and Incorporating Botanical Material

Botanical samples were collected and subsequently incorporated into the MG (João Murça Pires Herbarium, Museu Paraense Emílio Goeldi) (Ribeiro, O.D. 17:243,963 and Ribeiro, O.D. 18:243,964).

The fruit ripening process was monitored on a monthly basis at the collection sites. The onset of anthesis was identified (day 0), and the fruit was harvested during the final three phases of ripeness. These phases were determined in accordance with the morphological characteristics and ripening time of *P. macroloba* fruit and seeds, as outlined by Dantas et al. [10]. The fruit was collected at specific intervals corresponding to key developmental phases: 168 and 192 days post-anthesis (P1), 196 and 226 days post-anthesis (P2), and 241 and 270 days post-anthesis (P3). The determination of these intervals was based on the predominance of specific pericarp coloration, which served as a visual indicator of maturation. The collections were timed to coincide with the peak expression of each phenological phase.

**Figure 4:**
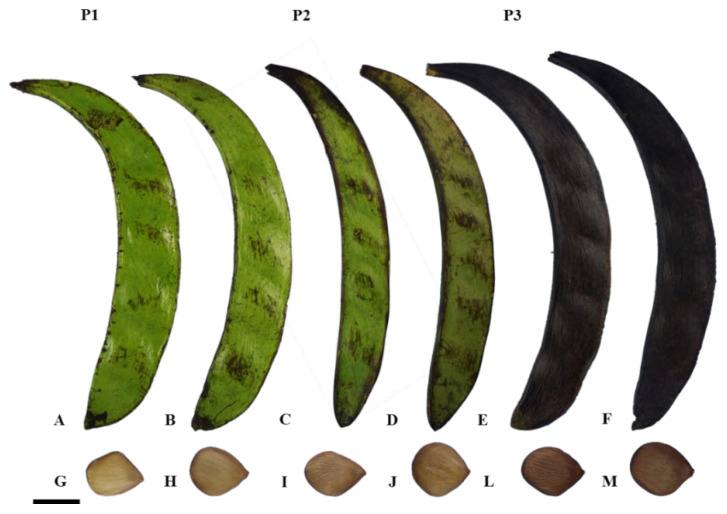
*Pentaclethra macroloba* (Willd.) Kuntze. P1: fruits with dark green pericarp: A and B and beige seeds with intensified brown color at the ends: G and H. P2: fruits with light green to yellow pericarp: C and D and light-brown seeds with dark ends: I and J. P3: fruits with completely black pericarp: E and F and dark brown seeds: L and M. Scale: 5 cm.

The fruit was collected when there was a predominance of fruits with a dark green pericarp and beige seeds with an intensification of the brown color at the ends (phase P1), light green to yellow pericarp and light-brown seeds with dark ends (phase P2), and fruits with a completely black pericarp and dark brown seeds (phase P3) (Figure 4). Subsequent to the collection process, the material was conveyed to the Propagules and Seedlings Biotechnology Laboratory (LBPM) of the Emílio Goeldi Museum of Pará, Belém, PA.

### 3.3. Seed Extraction

The procedure was conducted manually with the use of pruning shears, with the objective of eliminating the malformed seeds and those exhibiting signs of mechanical damage. Subsequently, the seeds were subjected to aseptic preparation, employing a solution of 1% sodium hypochlorite for a period of five minutes, followed by five consecutive washes with distilled water, in preparation for the subsequent tests [59].

### 3.4. Determining the Water Content of Seeds

The water content of the seeds was determined in accordance with the method outlined in the Rules for Seed Analysis (RAS) [59]. The analysis was performed using an oven set at 105 ± 3 °C, with a drying time of 24 h, and consisted of eight replicates, each containing two seeds. To optimize the evaporation process, the seeds were divided into four quadrants, ensuring uniform drying. After the drying period, the samples were removed from the oven and stored in desiccators until they reached room temperature. Thereafter, they were weighed, and the water content was calculated.

### 3.5. Vigor Test

The emergence test was conducted in an environment that lacked temperature and relative humidity control. The substrate utilized was a sterilized mixture of sand and tanned sawdust in a volumetric ratio of 1:1, with four replicates of 25 seeds each, in accordance with the methodology outlined by Ribeiro et al. [60]. Concurrent with the germination test, a daily evaluation was conducted over a 30-day period to record the number of emerged seedlings. The mean emergence time was calculated using the equation proposed by Edmond and Drapala [61], as outlined in Equation (1). The moment of seedling emergence was defined as the point at which the epicotyl surpassed the surface of the substrate, while germination was considered to have occurred when the seedlings had fully developed all their essential structures [60]. To assess the vigor of the seedlings, calculations were performed to evaluate the speed of emergence, as outlined in Equation (2) proposed by Maguire [62]. This analysis was complemented by a biometric analysis. This biometric analysis entailed the measurement of epicotyl length, neck diameter, and epicotyl dimensions in all seedlings at each phase of maturation [60]. A digital caliper and a millimeter ruler were used to ensure precision.

(1) $$AET=\frac{E_{1}*T_{1}+E_{2}*T_{2}+\cdots +E_{n}*T_{n}}{E_{1}+E_{2}+\cdots +E_{n}}$$

where

AET is defined as the average time required to reach maximum emergence in days;

E is calculated as the number of emergencies that occurred per day; and

T is the time in days.

(2) $$SEI=\frac{N_{1}}{D_{1}}+\frac{N_{2}}{D_{2}}+\cdots +\frac{N_{n}}{D_{n}}\;$$

where

SEI is the emergence speed index;

N is the number of seedlings verified on the day of the count; and

D is the number of days after sowing that the count was performed.

### 3.6. Analysis of Seed Physiological Quality and Seedling Vigor Data

The physiological quality of seeds across three maturation phases was analyzed statistically. Initially, the Levene test was employed to assess data homogeneity, followed by a series of normality tests to validate the parametric assumptions underlying the statistical analyses. The analytical data obtained on seed viability and seedling vigor were subjected to analysis of variance (ANOVA) and Tukey’s test in order to evaluate the dispersion of the measurements in relation to the mean. In cases where significant differences were identified, Tukey’s post hoc test was utilized to discern specific variations between treatments. Furthermore, Pearson’s linear correlation analysis was employed to examine the relationships between the variables.

The impact on the onset of emergence (ISE), average emergence time (AET), emergence speed index (SEI), seed emergence (E), ungerminated seeds (US), dead seeds (DS), abnormal seedlings (AS), seed germination (G), and epicotyl length (EL) was also evaluated. The seeds of *P. macroloba* were subjected to three treatments (P1, P2, and P3), and the following variables were analyzed: epicotyl diameter (ED), main root length (MRL), collar diameter (RCD), root dry mass (RDM), and aerial part dry mass (APDM) of the maturation phase.

Seedlings exhibiting structural development deficiencies, including underdeveloped or deformed roots, failure in the growth of the epicotyl and stem system, and malformations in the leaves, were considered abnormal [59]. These seedlings exhibited atypical development, deviating from the expected developmental trajectory for the species. Consequently, they were designated as abnormal.

The statistical analysis was conducted using the Minitab^®^ software (Minitab Statistical Software Inc., State College, PA, USA), version 17, and the PAST software [63], version 3.14. All statistical tests were performed with a significance level of *p* < 0.05.

### 3.7. Methods for Yield Determination and Chemical Analysis of Oils

#### Oil Extraction

In order to extract the vegetable oils, 100 g of seeds (equivalent to approximately 30 seeds) from the three phases of ripeness were utilized. The samples were ground with the seed coat intact. The seeds were ground to a particle size of 0.5 cm using a domestic blender. The vegetable oils were extracted via hydraulic pressing (15 tons) (SIWA, FM3), followed by a heat treatment at 80 °C for 30 min. The oils were stored in hermetically sealed amber glass bottles at a temperature of 4 °C in a refrigerator [64]. The lipid esterification of *P. macroloba* seeds was prepared in accordance with the methodology outlined by Khan and Scheinmann [65].

### 3.8. Analysis of the Chemical Composition of the Oils

The analysis of the fatty acids was conducted using gas chromatography equipment with a flame ionization detector (DIC) and gas chromatography coupled to mass spectrometry (GC-MS) at the Adolpho Ducke Laboratory (LAD/MPEG).

For GC-FID analysis, only one injection was performed based on the previous study published by our research group [50]. The analysis of fatty acid methyl esters was conducted using gas chromatography coupled with mass spectrometry (GC/MS) on a Shimadzu QP-2010 Plus system, employing a 30 m × 0.25 mm (diameter) × 0.25 μm (film thickness) DB-5ms fused silica capillary column coated with 5% diphenyl dimethylpolysiloxane. The following conditions were employed for the analysis: the injector temperature was set at 250 °C; the oven temperature was programmed to 100 °C for five minutes; the gradient was set at 5 °C/min to 260 °C for 20 min; helium was used as the carrier gas, with a linear speed of 36.5 cm/s (rate 1.2 mL min^−1^); the sample was injected in a splitless mode with 2 µL; electron impact ionization (EI) was set at 70 eV; and the ionization source and transfer line temperatures were set at 220 and 250 °C, respectively. The mass spectra were obtained via automatic scanning, encompassing mass fragments within the range of 35–400 Dalton.

The identification of the fatty acids present in the ion chromatograms was achieved through a comparative analysis of their mass spectra, including both molecular mass and fragmentation pattern, with those stored in the system library [66,67] and with data from the scientific literature. Furthermore, the retention indices of the identified fatty acids were also taken into account.

The quantification of the fatty acids was conducted using gas chromatography (GC) in a Shimadzu QP-2010 equipment (Shimadzu Corporation, Kyoto, Japan) equipped with a flame ionization detector (FID) under the aforementioned operating conditions, with the exception that the carrier gas was hydrogen.

A principal component analysis (PCA) was conducted on the fatty acid content to ascertain the similarities and differences between the ripening phases. The statistical analysis was conducted using Minitab^®^ (Minitab Statistical Software Inc., State College, PA, USA), version 17, and PAST [63] (version 3.14).

## 4. Conclusions

The maturation process of the fruits and seeds of *Pentaclethra macroloba* (Willd.) Kuntz has revealed that the physical and physiological quality of the seeds is influenced by the phase of harvest. The water content exhibited considerable variation, with the lowest levels observed in seeds derived from fruits that were approaching the point of natural dispersal. The color of the pericarp and seed coat serves as a reliable indicator of the optimal harvest time.

The study highlights that harvesting *P. macroloba* fruits at the P3 phase maximizes seed quality and oil yield, supporting sustainable extraction practices. It is, however, of interest to consider the collection of seeds at the ripening phase between P2 and P3, which is characterized by fruits with a yellowish-green pericarp and darkened areas, indicating the transition to P3. Furthermore, the seeds of these fruits also demonstrated a high oil yield following artificial desiccation.

These findings are particularly pertinent for extractive communities, which collect seeds after dispersal and are required to select the seeds, eliminate damaged ones, and remove the seed coat during oil extraction, a complex and time-consuming process. The distinct maturation phases did not impact the chemical composition of the extracted oils.

## Figures and Tables

**Table 1:** *Pentaclethra macroloba* (Willd.) Kuntze seeds at different maturation phases in Belém-PA ANOVA and Tukey tests for ISE-Days to initial seedling emergence, SEI-Mean time to emergence, AET-Emergence speed index, E-Seed emergence, US-Ungerminated seeds, DS-Dead seeds, AS-Abnormal seedlings, G-Seed germination, EL-Epicotyl length, MRL-Main root length, ED-Epicotyl diameter, RCD-Diameter of collar, APDM-Aerial part dry mass, and RDM-Dry mass of root.

	*F*	*p*	ω² **	Tukey		
				Phase 1	Phase 2	Phase 3
ISE	27.710	0.0001	0.8165	28 a *	25 a	17 b
SEI	60.350	6.11 × 10^−6^	0.9081	0.03 b	0.04 b	0.05 a
AET	52.110	1.13 × 10^−5^	0.8949	28.8 a	27.2 a	22.9 b
E	19.267	0.0006	0.7527	25 b	48 b	99 a
US	18.921	0.0006	0.7491	18.75 a	12.5 a	0.25 b
DS	3.000	0.1004	0.25	0 a	0.5 a	0 a
AS	3.550	0.0730	0.2978	0.5 a	0.75 a	1.5 a
G	16.174	0.0010	0.7166	23 b	45 b	93 a
EL	80.885	1.77 × 10^−6^	0.9301	4.31 b	8.46 b	30.23 a
MRL	1.321	0.3139	0.0508	11.16 a	11.54 a	13.65 a
ED	5.398	0.0288	0.4229	0.38 b	0.40 ab	0.48 a
RCD	47.114	1.71 × 10^−5^	0.8848	0.39 c	0.44 b	0.54 a
RDM	25.684	0.0002	0.8044	3.5059 b	3.9371 b	5.0147 a
APDM	46.617	1.78 × 10^−5^	0.8837	2.4206 c	4.0381 b	7.2676 a

* Different letters indicate significant differences between phases (Tukey, *p* < 0.05). ** Values close to 0 suggest a small or insignificant effect, while values close to 1 indicate a strong effect.

**Table 2:** *Pentaclethra macroloba* (Willd.) Kuntze seeds at different maturation phases in São Domingos do Capim-PA ANOVA and Tukey test for ISE-Days to initial seedling emergence, SEI-Mean emergence time, AET-Emergence speed index, E-Seed emergence, US-Ungerminated seeds, DS-Dead seeds, AS-Abnormal seedlings, G-Seed germination, EL-Epicotyl length, MRL-Main root length, ED-Epicotyl diameter, RCD-Diameter of collar, APDM-Aerial part dry mass, and RDM-Dry mass of root.

	*F*	*p*	ω² **	Tukey		
				Phase 1	Phase 2	Phase 3
ISE	40.793	3.07 × 10^−5^	0.8689	22 a *	19 b	12 c
SEI	119.884	3.26 × 10^−7^	0.9519	0.04 b	0.04 b	0.06 a
AET	111.401	4.48 × 10^−7^	0.9484	24 b	26 a	17 c
E	45.857	2.12 × 10^−5^	0.9193	40 b	66 b	99 a
US	44.966	2.07 × 10^−5^	0.8799	15 a	9 b	0.3 c
DS	***	***	***	***	***	***
AS	2.590	0.129	0.2095	1 a	1.25 a	0 a
G	52.047	1.13 × 10^−5^	0.8948	37 b	61 b	99 a
EL	80.590	1.80 × 10^−6^	0.9299	6.49 b	7.70 b	33.80 a
MRL	20.780	0.0004	0.7672	10.69 b	12.56 b	19.08 a
ED	7.270	0.0130	0.511	0.42 b	0.48 ab	0.57 a
RCD	13.130	0.0020	0.669	0.47 b	0.51 b	0.62 a
RDM	100.96	6.85 × 10^−7^	0.9433	3.7544 c	4.6356 b	6.3029 a
APDM	212.46	2.66 × 10^−8^	0.9724	3.7318 b	4.5687 b	10.8596 a

* Different letters indicate significant differences between phases (Tukey, *p* < 0.05). ** Values close to 0 suggest a small or insignificant effect, while values close to 1 indicate a strong effect. *** = Missing values.

**Table 3:** Seed yield, fixed oil content, and fixed oil yield of *Pentaclethra macroloba* (Willd.) Kuntze seeds have three maturity phases.

Place	Phase	Fixed Oil Content (g)	Fixed Oil Content (%)	Code
Belém	1	3.590	3.6	B1
Belém	2	8.710	8.7	B2
Belém	3	12.818	12.8	B3
SDC	1	1.800	2.8	SDC1
SDC	2	6.503	7.5	SDC2
SDC	3	13.139	13.1	SDC3

Legend: Location and maturity phase—(B1, B2, B3: seeds from Belém at maturity phases 1, 2, and 3, respectively); (SDC1, SDC2, SDC3: seeds from São Domingos do Capim at maturity phases 1, 2, and 3, respectively).

**Table 4:** Fatty acid compositions of oils extracted from *Pentaclethra macroloba* (Willd.) Kuntze seeds stored in three maturation phases.

Fatty Acid, Methyl Ester	Symbolism	B1	B2	B3	SDC1	SDC2	SDC2
Palmitic acid	C16:0	1.81	1.76	1.59	2.25	2.07	2.07
Linoleic acid	C18:2 (9,12) ω6	8.45	7.92	6.68	5.65	7.59	5.44
Oleic acid	C18:1 (9) ω9	54.91	54.5	65.23	49.1	52.7	51.7
Stearic acid	C18:0	3.21	3.13	3.12	4.32	5.07	4.75
Gondoic acid	C20:1 (11)	1.58	1.6	1.13	2.05	1.56	2.09
Arachidic acid	C20:0	0.83	0.86	0.72	1.22	1.36	1.37
Erucic acid	C22:1 (13) ω9	0.65	0.67	0.32	0.98	0.59	1.03
Behenic acid	C22:0	16.02	16.38	13.24	17.71	17.17	17.22
Tricosylic acid	C23:0				0.13	0.07	0.12
Nervonic acid	C24:1 (15)				0.13		0.17
Lignoceric acid	C24:0	12.29	12.81	7.97	15.85	11.41	13.5
Cerotic acid	C25:0	0.25	0.28		0.61	0.26	0.47

Legend: Location and maturity phase—(B1, B2, B3: seeds from Belém at maturity phases 1, 2, and 3, respectively); (SDC1, SDC2, SDC3: seeds from São Domingos do Capim at maturity phases 1, 2, and 3, respectively).

## Data Availability

Data is contained within the article.
